# Genomic disruption of the histone methyltransferase *SETD2* in chronic lymphocytic leukaemia

**DOI:** 10.1038/leu.2016.134

**Published:** 2016-06-10

**Authors:** H Parker, M J J Rose-Zerilli, M Larrayoz, R Clifford, J Edelmann, S Blakemore, J Gibson, J Wang, V Ljungström, T K Wojdacz, T Chaplin, A Roghanian, Z Davis, A Parker, E Tausch, S Ntoufa, S Ramos, P Robbe, R Alsolami, A J Steele, G Packham, A E Rodríguez-Vicente, L Brown, F McNicholl, F Forconi, A Pettitt, P Hillmen, M Dyer, M S Cragg, C Chelala, C C Oakes, R Rosenquist, K Stamatopoulos, S Stilgenbauer, S Knight, A Schuh, D G Oscier, J C Strefford

**Affiliations:** 1 Academic Unit of Cancer Sciences, Faculty of Medicine, Cancer Research UK Centre and Experimental Cancer Medicine Centre, University of Southampton, Southampton, UK; 2 Oxford National Institute for Health Research, Biomedical Research Centre/Molecular Diagnostic Centre, University of Oxford, Oxford, UK; 3 Department of Internal Medicine III, Ulm University, Ulm, Germany; 4 Centre for Biological Sciences, Faculty of Natural and Environmental Sciences, University of Southampton, Southampton, UK; 5 Centre for Molecular Oncology, Barts Cancer Institute, Queen Mary's, University of London, London, UK; 6 Department of Immunology, Genetics and Pathology, Science for Life Laboratory, Uppsala University, Uppsala, Sweden; 7 Department of Molecular Pathology, Royal Bournemouth Hospital, Bournemouth, UK; 8 Institute of Applied Biosciences, Center for Research and Technology Hellas, Thessaloniki, Greece; 9 Department of Haematology, University Hospital of Salamanca-Biomedical Research Institute of Salamanca, IBMCC, Comprehensive Cancer Center Research, University of Salamanca-CSIC, Salamanca, Spain; 10 Department of Haematology, Altnagelvin Area Hospital, Western Health and Social Care Trust, Londonderry, UK; 11 Department of Molecular and Clinical Cancer Medicine, Royal Liverpool and Broadgreen University Hospitals NHS Trust, Liverpool, UK; 12 Department of Haematology, St James's University Hospital, Leeds, UK; 13 College of Medicine, Biological Sciences and Psychology, University of Leicester, Leicester, UK; 14 Division of Hematology, Department of Internal Medicine, The Ohio State University, Columbus, OH, USA

## Abstract

Histone methyltransferases (HMTs) are important epigenetic regulators of gene transcription and are disrupted at the genomic level in a spectrum of human tumours including haematological malignancies. Using high-resolution single nucleotide polymorphism (SNP) arrays, we identified recurrent deletions of the *SETD2* locus in 3% (8/261) of chronic lymphocytic leukaemia (CLL) patients. Further validation in two independent cohorts showed that *SETD2* deletions were associated with loss of *TP53*, genomic complexity and chromothripsis. With next-generation sequencing we detected mutations of *SETD2* in an additional 3.8% of patients (23/602). In most cases, *SETD2* deletions or mutations were often observed as a clonal event and always as a mono-allelic lesion, leading to reduced mRNA expression in *SETD2*-disrupted cases. Patients with *SETD2* abnormalities and wild-type *TP53* and *ATM* from five clinical trials employing chemotherapy or chemo-immunotherapy had reduced progression-free and overall survival compared with cases wild type for all three genes. Consistent with its postulated role as a tumour suppressor, our data highlight *SETD2* aberration as a recurrent, early loss-of-function event in CLL pathobiology linked to aggressive disease.

## Introduction

The transfer of methyl groups from *S*-adenosyl methionine to lysine or arginine residues on histone proteins, catalysed by histone methyltransferases (HMTs), is an important regulator of gene transcription. Accordingly, HMTs are disrupted by various mechanisms, including chromosomal translocations, genomic loss and/or point mutations in both solid and haematological malignancies.^1^ Among the increasing number of HMT aberrations identified in human malignancies, recurrent loss and/or inactivating mutations of the tumour suppressor gene *SETD2* were initially identified in clear cell renal cell carcinoma^2^ and subsequently in other solid tumours, for example, high-grade gliomas.^3^ Moreover, *SETD2* mutations have been reported in a subset of patients with acute lymphoblastic leukaemia^4^ and acute myeloid leukaemia, especially those with rearrangements in the another HMT gene, *MLL*.^5^ SETD2 is the only enzyme that catalyses the trimethylation of lysine 36 on histone 3 (H3K36me3), one of the major chromatin marks associated with active transcription. Recent studies have linked SETD2 to the maintenance of genomic integrity through coordination of homologous recombination repair after double strand breaks. The loss of SETD2 impairs DNA repair and enhances genomic instability, supporting its tumour suppressor role.^6, 7, 8, 9^

Chronic lymphocytic leukaemia (CLL) is characterized by remarkable clinical heterogeneity such that some patients pursue an indolent course while others require early treatment. Considerable effort has focused on understanding the genetic diversity that underpins this clinical heterogeneity. High-resolution genomic arrays and next-generation sequencing have identified recurring novel regions of genomic copy-number aberrations (CNAs) like del(13q), del(11q), trisomy 12 and del(17p) and recurrent driver mutations in genes such as *TP53*, *ATM*, *SF3B1* and *NOTCH1*, respectively (reviewed in Guièze and Wu^10^). Mutations frequently involve genes encoding proteins with important roles in cell signalling, cell cycle control, DNA repair and RNA splicing and processing; however the reported incidence of mutations in chromatin modifiers is lower than in many other haematological malignancies.

In this study, we report the identification of recurrent deletions and mutations of the *SETD2* gene in large, well-characterized CLL cohorts. *SETD2* lesions appear to represent early events in CLL pathogenesis, often coexisting with, but preceding *TP53* abnormalities. They are associated with genomic complexity and chromothripsis, and identify a subgroup of patients with poor outcome.

## Patients and methods

### Patients

We studied samples taken from 1006 CLL patients either at entry into one of five clinical trials or from a cohort of untreated patients with progressive disease managed at the Royal Bournemouth Hospital. Four randomized trials (ADMIRE, ARCTIC, UK CLL4^(ref. 11)^ and GCSG CLL8^(ref. 12)^) compared chemo or chemo-immunotherapy regimens in fit previously untreated patients while the fifth trial (SCSG CLL2O) enroled ultra-high-risk patients who were either refractory to a purine analogue or were previously untreated with a 17p deletion. Further details of the clinical trials are provided in Supplementary Table S1. All patients were diagnosed using standard morphologic and immunophenotypic criteria. Informed consent was obtained from all patients in accordance with the Helsinki declaration, and this study was approved by national or regional research ethics committees.

Patients were grouped into three cohorts (discovery (*n*=261), extension (*n*=635) and ultra-high-risk (*n*=110)); details of the cohort composition and *SETD2* analysis are summarized in Table 1, Supplementary Methods and Supplementary Figure S1. DNA was extracted from CLL B-cell samples (all with >80% tumour purity) and from matched germline DNA for *SETD2*-mutated cases as outlined in Supplementary Methods. The assessment of established biomarkers was performed as previously described.^13^ In total, 572 and 602 samples were screened for *SETD2* loss and mutation, respectively, with 168 cases screened for both loss and mutation.

### Genome-wide microarray-based copy-number analysis

DNA from 261 discovery and 110 ultra-high-risk cases was amplified, labelled and hybridized to the Affymetrix SNP6.0 platform, aligned onto the human genome sequence (GRCh37) and analysed in Partek Genomics Suite (Partek, St Louis, Inc., MO, USA) as reported previously.^14, 15, 16, 17, 18^ DNA from 201 pre-treatment extension cases (ADMIRE and ARCTIC) was hybridized to the Illumina HumanOmni1-Quad and HumanOmniS-8 platforms according to the manufacturer's protocols.^19, 20^ Further experimental details are provided in the Supplementary Methods.

### Targeted re-sequencing and whole-exome sequencing

Ninety-three CLL samples from the discovery cohort (and five matched germline controls) were processed and analysed for mutations in *SETD2* (all exons) and a number of clinically relevant genes with a bespoke Haloplex Target Enrichment system (Agilent Technologies, Santa Clara, CA, USA) (Supplementary Methods and Table 1) and processed and analysed as previously reported.^21^ An additional 231 cases from our pre-treatment extension cohort were screened for *SETD2* mutations using a TruSeq Custom Amplicon panel (Illumina Inc., San Diego, CA, USA) as previously described.^20, 22^ All the variants identified by both platforms were annotated against dbSNP (build 135) and functional prediction was also performed using SIFT andPolyphen2 analysis. Somatically acquired *SETD2* mutations (*n*=4) were also identified in the recent whole-exome sequencing study of 278 matched tumour and germline cases from the GCSG CLL8 study.^23^ Additional experimental details are provided in the Supplementary Methods.

### Sanger validation

Variants in *SETD2* were subjected to validation by conventional Sanger-based sequencing of PCR products obtained from tumour (*n*=11) and where possible, paired normal genomic DNA (*n*=5). The expression of *SETD2* mutations at mRNA level was also tested in samples with available material (*n*=4). Primers for DNA or mRNA validation are listed in Supplementary Table S2.

### Quantitative reverse transcriptase-PCR

Total RNA was isolated from purified CLL cells of 36 patient samples using RNeasy columns (Qiagen, Manchester, UK) and reversed transcribed using the Improm II RT-PCR kit (Promega, Southampton, UK) according to the manufacturer's instruction. Primers and probes for the housekeeping genes (18 s) and target genes (*CCDC12, NBEAL2, KIF9, KLHL18, SETD2*) were selected using the Universal Probe Library (Roche Applied Science, Burgess Hill, UK) (Supplementary Table S3). Two independent assays were designed to ascertain expression of 3′ and 5′ *SETD2*. Normal B-cell mRNA was use to normalize the expression of each gene by delta-delta CT method as previously described.^24^

### Statistical analysis

Statistical analysis was performed with SPSS v22. Differences between samples were analysed by Mann–Whitney *U*-test. Progression-free survival (PFS) and overall survival (OS) were calculated for clinical trial samples from randomization. Survival analysis was performed by Kaplan–Meier and log-rank analysis. Significant differences were considered with *P*-values lower than 0.05.

## Results

### Recurrent deletions of 3p are a feature of CLL

**Figure 1:**
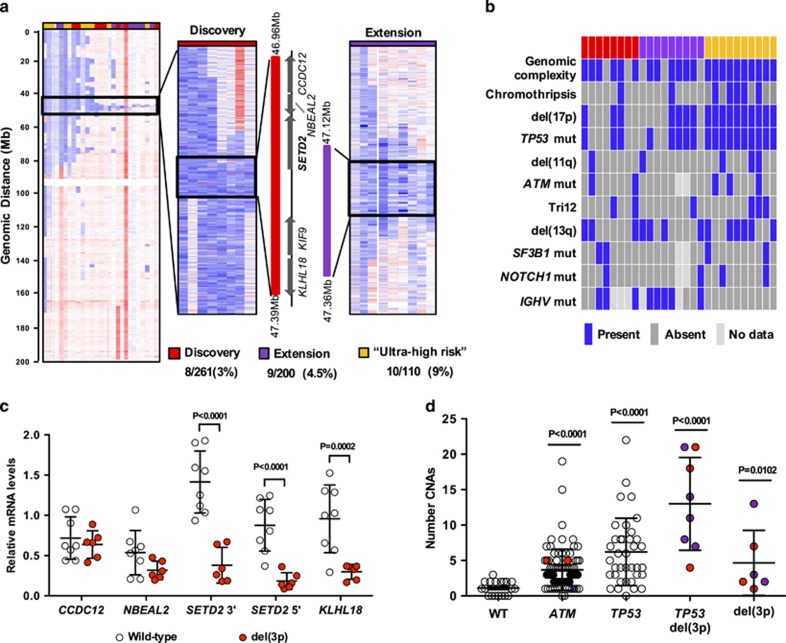
*SETD2* deletions in our discovery, extension and ultra-high-risk cohorts. (**a**) SNP6.0 data for the del(3p) cases. Genomic location is indicated by the ladder to the left. Each column represents one patient. Loss, gain and normal copy number are shown as blue, red and white, respectively. The black box indicates the MDR, and is displayed in greater detail for our discovery and extension cohorts. The genes in the MDR with their transcriptional direction are displayed in the middle, with the MDR from the discovery and extension cohorts shown by the red and purple bars, respectively. (**b**) Matrix displaying the biomarkers and genomic features associated with del(3p) cases with the discovery, extension and ultra-high-risk cases shown in red, purple and yellow, respectively. (**c**) Real-time PCR expression for the five genes localized in the discovery MDR in cases with or without del(3p). All the samples were negative for KIF9. 18 s was employed as housekeeping gene. Expression in normal B-cells was used as a normalization sample. Mean±s.d. is represented. (**d**) Scatterplots displaying the number of CNA observed in subgroups of our cohort (excluding ultra-high-risk cases). Cases were assigned to a subgroup using a hierarchical model; presence of del(17p) and/or *TP53* mutation, then del(11q) and/or *ATM* mutation, then del(3p) cases with and without *TP53* abnormalities and then wild-type (WT) cases containing no del(17p), del(11q), del(3p) or mutations in *ATM* and *TP53*. Mean±s.d. is represented.

We identified 1024 acquired CNAs (mean 3.9, range 0–45) in our discovery cohort (Supplementary Table S4). Deletions of chromosome 3p [del(3p)] were observed in eight patients (3%), ranged from 0.45 to 81 Mb in size (Supplementary Table S5) and identified a well delineated minimally deleted region (MDR) between genomic location 46.96 and 47.39 Mb, containing the genes *CCDC12, NBEAL2, SETD2, KIF9* and *KLHL18* (Figure 1a). We compared the expression of these genes by quantitative reverse transcriptase-PCR in 3p deleted (*n*=6) versus non-3p deleted patients (*n*=8) (Figure 1c). We were not able to detect the expression of *KIF9* mRNA in CLL or normal B-cells. Within the MDR, the HMT gene *SETD2* was significantly underexpressed, measuring by two different assays targeting the 3′ or 5′ region of the mRNA (*P*<0.0001 for both assays).

We then aimed to confirm the presence of 3p deletions and refine the MDR in our extension cohorts. Firstly, we identified nine del(3p) cases (4.5%) in our extension pre-treatment cohort, permitting the MDR to be refined to the *SETD2* and *KIF9* loci (47.12–47.36 Mb; Figure 1a and Supplementary Table S5). Across our discovery and pre-treatment extension cohorts, *SETD2* deletions were present in 17/461 cases (3.7%), significantly associated with deletions and/or mutations of *TP53* (*P*=0.003) and genomic complexity (⩾3 deletions,^25^ *P*=0.04) (Figure 1b). GISTIC 2.0 analysis,^26^ an algorithm for identifying statistically significant regions of CNA above an estimated background rate (FDR *q*-value <0.25), showed that in 39 *TP53* deleted cases (del(3p), *n*=15), the *SETD2* region on 3p21.31 was deleted at a significant frequency (*q*-value=0.001), ranked third after del(13q) and del(17p) (Supplementary Figure S2).

Interestingly, *SETD2* deletions without concomitant *TP53/ATM* abnormalities (*n*=6) also exhibited significantly more genomic complexity than wild-type patients (*P*=0.01; Figure 1d). Two *SETD2*-deleted cases showed evidence of chromosome 3 chromothripsis (based on >10 CNAs per chromosome^18^) (Figures 1a and b). In the ultra-high-risk cohort, *SETD2* deletions were detected in 9% of cases (10/110), and were significantly enriched compared with the pre-treatment cohort (*P*=0.009). All 10 had loss of *TP53* and 5 had concomitant chromosome 3 chromothripsis (Figure 1b). To further establish the significance of our *SETD2* deletion in cases with chromosome 3 chromothripsis, we mapped all recurrently 3p deletions in these cases. This analysis showed that while additional regions of recurrent deletion were observed on 3p, the only regions shared across all patients included the *SETD2* locus (Supplementary Table S6).

We analysed *SETD2* expression in an extended cohort of patients with 3p deletions (*n*=16), and again the expression was diminished in these patients compared with wild-type patients (*P*=0.0068; Supplementary Figure S3). In order to study the clonal nature of the *SETD2* deletions, we assigned each genomic CNA with a relative copy-number value by normalizing CNA intensity values from array features. We excluded regions with gain and sex chromosome CNAs from the analysis. The cutoff for normal copy number was established between 1.7 and 2.3. We could infer that the 3p deletion was in the dominant clonal population in 11/18 (61%) cases with data available for analysis (Supplementary Figure S4).

### *SETD2* mutations in CLL

**Figure 2:**
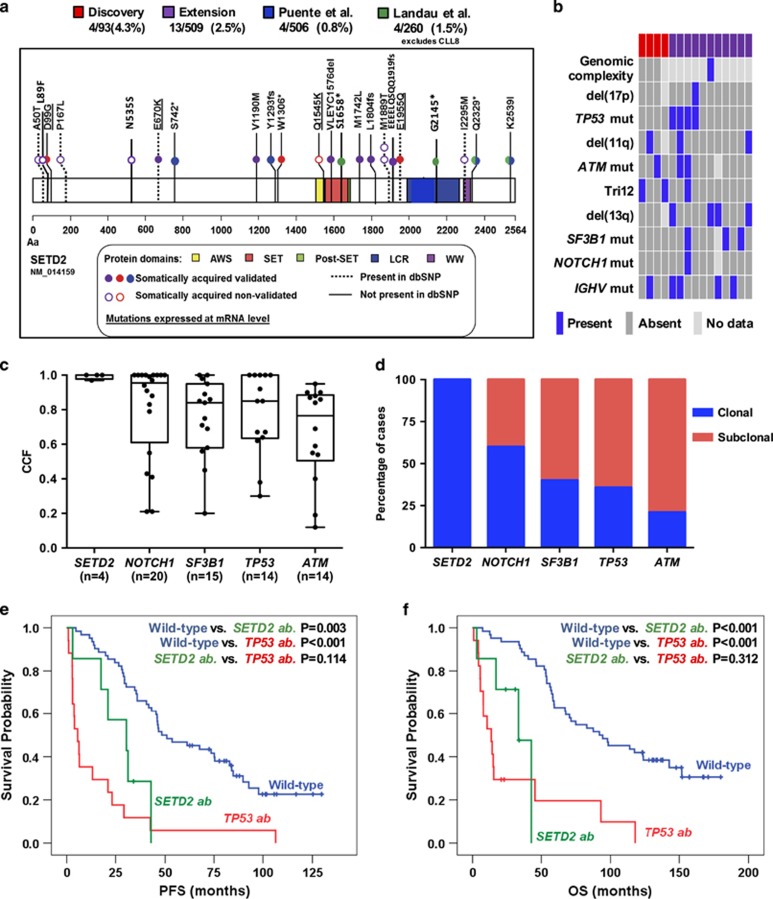
*SETD2* mutations in our discovery and extension cohorts. (**a**) Schematic diagram of the Setd2 protein with their key functional domains. Mutations are displayed in the diagram. The colour denotes the cohort, and the filled circles are mutations that have been confirmed as somatically acquired. (**b**) Matrix displaying the biomarkers and genomic features associated with *SETD2*-mutated cases in the discovery (red) and extension (purple) cases. (**c**) Analysis of the clonality for *SETD2* and other recurrently mutated genes on CLL. For each case the cancer cell fraction (CCF) is derived manually or with the ABSOLUTE algorithm. Only somatically acquired validated mutations are displayed (cases 69, 100, S21 and 88). The number of mutations (*n*) for each gene in the analysis is shown (bottom). (**d**) Percentage of cases harbouring clonal or subclonal mutations for each of the genes displayed. (**e**) Kaplan–Meier and log-rank analysis for progression-free survival (PFS) in patients carrying *SETD2* abnormalities (‘*SETD2* ab') but wild type for *TP53* or *ATM* deletion and/or mutation compared with those with TP53 abnormalities (‘*TP53* ab') and those wild type for *TP53*, *ATM* and *SETD2* (‘wild type'). (**f**) Kaplan–Meier and log-rank analysis for overall survival (OS) in the same categories described in (**e**).

To identify somatic gene mutations, we initially employed targeted re-sequencing of 93 discovery cohort cases and identified 122 non-silent mutations (non-synonymous *n*=80, frameshift indel *n*=20, splicing *n*=9, nonframeshift indel *n*=6, stopgain *n*=6, stoploss *n*=1) targeting 37 genes in 71/93 cases (mean 1.8, range 1–4). Sanger sequencing confirmed 93.6% of the tested variants (*n*=79), while the remaining unconfirmed variants were present at low read depth (*n*=3) or in a low percentage of mutant reads (*n*=2). We found *ATM* (*n*=14), *TP53* (*n*=14), *NOTCH1* (*n*=20) and *SF3B1* (*n*=15) mutations at a frequency expected for the studied cohort, which aligns with published data and demonstrates the validity of the re-sequencing platform. We identified non-synonymous *SETD2* mutations in four (4.3%) discovery cases (p.D99G, p.Q1545K, p.W1306*, p.E1955Q) (Figures 2a and b). Sanger sequencing validated that all of the *SETD2* mutations were present in tumour DNA. We obtained matched germline DNA from three patients and confirmed that the mutations were somatically acquired (p.D99G, p.W1306*, p.E1955Q) (Supplementary Figure S5A).

To corroborate this preliminary observation, we investigated 231 cases of our pre-treatment extension cohort by TruSeq amplicon-based sequencing. We identified an additional nine (3.9%) *SETD2* mutations (p.A50T, p.L89F, p.P167L, p.N535S, p.E670K, p.M1742L, p.M1889T (x2), p.I2295M) (Figures 2a and b). Sanger sequencing confirmed each *SETD2* variant in the tumour material and in two cases with germline material available, the variants were somatically acquired. Assessment of whole-exome sequencing data of the CLL8 study^23^ samples included in our pre-treatment extension cohort revealed the presence of somatically acquired *SETD2* mutations in 4/278 cases (1.4%), namely, p.EEEELQSQQ1919fs, p.L1804fs, p.VLEYC1576del, p.V1190M. None of these *SETD2* mutations (Table 2) are annotated in COSMIC.^27^ During the preparation of this manuscript, a study performed by Puente *et al.*^28^ in 506 CLL patients also described both *SETD2* mutations (0.8% of cases) and deletions in 3p (2% of cases) whose MDR encompassed *SETD2* while Landau *et al.*^23^ identified *SETD2* mutations in 8/538 (1.5%) cases.

In total, across our cohorts there were 15 somatically acquired *SETD2* variants (15/602; 2.5%). An additional eight variants that could not be examined in germline material were either absent (*n*=3), reported to have a very low prevalence (*n*=5) in 1000 Genomes project or have a subclonal variant allele frequency (%VAF <0.45 (*n*=1); Table 2). Therefore, while these eight variants are predicted to be functionally deleterious, we cannot exclude that the minority may be rare germline variants as they exhibit clonal variant allele frequencies in the tumour material.

We were able to confirm the expression of the *SETD2* mutations at mRNA level in four of our patients with available material (p.D99G, p.Q1545K, p.E1955Q, p.E670K) (Supplementary Figure S5A), and quantiative reverse transcriptase-PCR analysis of three *SETD2* mutated samples showed that *SETD2* mRNA expression was reduced compared with wild-type patients (*P*=0.035; Supplementary Figure S3).

We performed integrative analysis of 93 cases from our discovery cohort with Haloplex re-sequencing and SNP6.0 copy-number data available, by employing the ABSOLUTE algorithm.^29^ This approach estimates the cancer cell fraction harbouring a given mutation by correcting for sample purity and local copy-number changes. Mutations were classified as clonal if the cancer cell fraction was >0.95 with a probability >0.5 and subclonal otherwise.^30^ In additional cases with proven-somatic *SETD2* mutations (*n*=4) and paired copy-number data from our pre-treatment validation cohorts, we performed this estimation by manually correcting for tumour sample purity and local copy number. Our analysis demonstrated the expected subclonal distribution of established gene mutations, such as *TP53*, *ATM*, *SF3B1* and *NOTCH1*. Interestingly, all our somatically acquired *SETD2* mutations exhibited a clonal cancer cell fraction, suggesting that these mutations may be early events in the evolution of CLL (Figures 2c and d and Supplementary Figures S5B and C), although further studies are required to confirm this observation.

### *SETD2* aberrations are associated with inferior progression-free and OS

Finally, we analysed the impact of *SETD2* abnormalities (deletion or somatically acquired mutation) on PFS and OS in front-line trial patients. We observed a significantly shorter PFS in cases with *SETD2* abnormalities that were wild type for *TP53/ATM* (*n*=7), compared with cases wild type for *TP53/ATM/SETD2* (*n*=62) (PFS: 30 vs 48 months; *P*=0.003) (Figure 2e). The same patients with *SETD2* abnormalities (*n*=7) also had a shorter OS than wild-type patients (*n*=62) (OS: 34 vs 92 months; *P*<0.001) (Figure 2f). While these data suggest that *SETD2* aberration may be clinically relevant, further investigation in larger cohorts is needed to understand their full impact on survival.

## Discussion

This study was based on an initial high-resolution SNP6.0 array analysis of 261 untreated patients with progressive CLL, which identified a recurrent deletion of the short arm of chromosome 3 in 3% of cases (*n*=8). The MDR included the *CCDC12, NBEAL2, SETD2, KIF9* and *KLHL18* genes, of which *SETD2* was the most significantly underexpressed in tumour cells. We then identified clonal, somatically acquired *SETD2* mutations in 4.3% of this cohort; no mutated case had a concomitant *SETD2* deletion.

The *SETD2* gene encodes a 230 kDa protein that is non-redundantly responsible for all trimethylation of lysine 36 on histone H3 (H3K36me3),^31, 32^ a mark that is associated with actively transcribed regions and is involved in transcriptional elongation and splicing.^33^ In addition, recent studies have linked this epigenetic histone mark to other important cellular processes such as the regulation of mismatch repair, efficient homologous recombination and the maintenance of genomic stability.^7, 8, 9^ *In vitro* inhibition of Setd2 decreases global levels of H3K36me3 and impairs the recruitment of the mismatch recognition protein hMutSα onto chromatin, thereby preventing appropriate DNA mismatch repair. Cells lacking the Setd2 protein display microsatellite instability and have elevated levels of spontaneous mutations.^7, 34, 35, 36^ Inactivating *SETD2* mutations were first described in clear cell renal cell carcinoma,^2, 6^ subsequently in other solid tumours such as paediatric high-grade gliomas and most recently in a subset of patients with acute lymphoid and myeloid leukaemias.^2, 5, 37, 38^ *SETD2* mutations in clear cell renal cell carcinoma are frequently associated with 3p deletions resulting in loss of both *SETD2* and VHL genes, while in acute leukaemias, *SETD2* mutations may be bi-allelic but 3p loss is rare. *SETD2* genomic abnormalities are associated with decreased H3K36me3 levels, a distinctive DNA methylation signature^6^ and chemoresistance in paediatric acute lymphoblastic leukaemia.^39^ In MLL-rearranged cells from acute leukaemic patients, Setd2 knockdown is implicated in disease initiation and progression by promoting the self-renewal capacity of leukaemic stem cells.

In view of the role of *SETD2* disruption in tumorigenesis and the identification of *SETD2* abnormalities in our discovery cohort, we then accrued samples from other patient cohorts, including the GCLLSG CLL8 cohort in which 3p deletions had also been detected,^18^ to confirm the incidence of *SETD2* disruption and evaluate its biological and clinical consequences in CLL. Previously untreated patients sampled at randomization to chemo or chemo-immunotherapy trials had a similar incidence of 3p deletions (4.5%) to that seen in the discovery cohort while a higher incidence of loss (9%) was found in the ultra-high-risk cohort. The inclusion of additional cohorts enabled a smaller MDR to be defined, including *SETD2* and *KIF9*, implicating *SETD2* as the key deleted gene. The incidence of *SETD2* mutations was comparable in all cohorts tested, no synonymous mutations were identified and when germline material was tested, all mutations were somatically acquired. The diverse sequencing strategies utilized in this current study precluded the application of computational tools like MutSigCV,^40^ an algorithm that identifies significantly mutated genes by accounting for background mutation rate, DNA replication time and the gene size. However, we did assess the *SETD2* background mutation rate, expression level and replication timing data from Lawrence *et al.*^40^ demonstrating that *SETD2* shares no properties associated with false-positive candidate cancer genes (Supplementary Figure S7). The recent studies by Puente *et al.* and Landau *et al.* published during the preparation of this manuscript confirm the rare but recurrent nature of *SETD2* abnormalities.^23, 28^ *SETD2* deletions were not over-represented by analysis of whole-exome sequencing generated copy-number data in the work by Landau and the mutation frequencies of both studies were lower than those in our study. The different frequencies reported in these two studies could be explained by cohort composition, as our study included ultra-high-risk CLL and patients randomized to clinical trials.

As we found *SETD2* mRNA expression to be downregulated in cases with either *SETD2* deletion or mutations and as we did not observe bi-allelic *SETD2* abnormalities, we assessed whether *SETD2* may also be deregulated by DNA methylation. Kulis and co-workers^41^ reported no differential methylation levels in the *SETD2* gene body and promoter regions (15 and 9 CpG probes), respectively, between unmutated or mutated CLL or major cytogenetic subtypes and *SETD2* mRNA expression was not correlated with gene methylation status (doi:10.1038/ng.2443: Supplementary Tables S5 and S11).^41^ Preliminary analysis of our own unpublished Illumina 450 K methylation array data also demonstrated no differences between mutated and unmutated CLL for these probes (Supplementary Figure S6). In addition, when we analysed *SETD2* expression in a published CLL data set (http://www.ncbi.nlm.nih.gov/geo/query/acc.cgi?acc=GSE2466)^44^ using the Oncomine portal (https://www.oncomine.org),^42^ we observed a heterogeneous pattern. Reduced levels are evident in the minority of patients, which given our observed association between *SETD2* deletion and expression could imply gene deletion in those Oncomine samples with low mRNA expression. Together, this suggests that DNA methylation does not play a substantial role in regulating *SETD2* expression in B-CLL cells, as previously noted in acute leukaemia.^5^

Across all cohorts, *SETD2* deletion was found in both *IGHV* mutated and unmutated cases but was strongly associated with *TP53* loss and mutation, likely accounting for its higher incidence in the ultra-high-risk cohort. We also noted an association with genomic complexity even in cases lacking a *TP53* or *ATM* abnormality, consistent with the role of *SETD2* in maintaining genomic stability. Moreover, we identify several *SETD2* deletions that appeared to be the result of chromothripsis. The somatically acquired *SETD2* mutations showed a comparable genomic distribution to those previously described in other tumours and were predicted to have deleterious functional consequences. Furthermore, their association with significantly reduced mRNA expression in those cases analysed, suggest that they either directly affect mRNA expression or coexist with other defects in transcriptional control at this locus. Interestingly, we did not observe a statistically significant association between *SETD2* mutations and *TP53* abnormalities or genomic complexity, the implication of which may be differing functional consequences of mono-allelic loss and mutation.

In our study, both *SETD2* deletions and mutations often appeared to be clonal and may precede *TP53* abnormalities in at least some cases. Setd2 has been shown to directly regulate the transcription of a subset of genes via cooperation with the transcription factor p53,^43^ and the link between *SETD2* and *TP53* is an interesting association worthy of functional validation. It is possible that the *SETD2* alterations present in our CLL cases may contribute to further inactivation of p53-mediated checkpoint control, a situation that has been proposed in clear cell renal cell carcinoma.^8^ The low frequency of *SETD2* disruption and the association with *TP53* abnormalities hinder an accurate assessment of its clinical consequences. Nevertheless, we observed a shorter PFS and OS in patients with *SETD2* but no *TP53* or *ATM* abnormalities compared with cases wild type for all three genes. In support of this preliminary clinical observation, it has been shown that 3p deletions in head and neck squamous carcinoma are associated with reduced survival.^44^ Furthermore, the authors showed that the coexistence of a *TP53* abnormality with del(3p) decreased survival further, an observation that we could not confirm in our cohort.

In summary, our current study provides the first comprehensive analysis of CNAs and mutations targeting the *SETD2* gene in a large cohort of patients with CLL. We find somatic deletions and mutations in ~7% of CLL patients requiring treatment. These associate with *TP53* dysfunction, genomic complexity and chromothripsis and may be early clonal events. Functional studies are now warranted to elucidate the exact biological importance of *SETD2* in CLL pathogenesis, but our data add to a growing body of evidence suggesting a role for H3K36me3 in tumorigenesis that may be exploited for the development of novel therapeutic approaches.

## Figures and Tables

**Table 1:** Cohort characteristics

*Characteristics*	*Discovery*, N *(%)*	*Extension*, N *(%)*	*Ultra-high-risk*, N *(%)*
Number of cases (number with germline material)	261 (5)	635 (280)	110
Origin	CLL4/Local cohort	CLL4/ARCTIC/ ADMIRE/CLL8	CLL2O

*Treatment naïve*			
Yes	261 (100)	635 (100)	35 (32)
No	—	—	75 (68)

*Gender*			
Male	192 (74)	336 (53)	79 (72)
Female	69 (26)	299 (47)	31 (28)

*IGHV* gene mutational status			
Unmutated	120 (46)	350 (55)	96 (87)
Mutated	68 (26)	232 (37)	12 (11)
No data	73 (28)	53 (8)	2 (2)

*del(17p)*			
Yes	29 (11)	32 (5)	88 (80)
No	214 (82)	565 (89)	22 (20)
No data	18 (7)	38 (6)	—

*del(11q)*			
Yes	79 (30)	138 (22)	21 (19)
No	165 (63)	461 (73)	89 (81)
No data	17 (7)	36 (5)	—

*Tri12*			
Yes	23 (9)	58 (9)	19 (17)
No	199 (76)	418 (66)	91 (83)
No data	39 (15)	159 (25)	—

*Del(13q)*			
Yes	103 (40)	252 (40)	65 (59)
No	63 (24)	224 (35)	45 (41)
No data	95 (37)	159 (25)	—
Chromothripsis	8 (3)	9 (1.4)	10 (9)
*SETD2* deleted	8/261 (3)	9/201 (4.5)	10/110 (9)
*SETD2* mutated	4/93 (4.3)	11/509 (2.2)	—
*SETD2* deleted with *TP53* abnormalities	3/8 (37.5)	5/9 (55.5)	10/10 (100)
*SETD2* mutated with *TP53* abnormalities	0/4 (0)	4/11 (36.4)	—

**Table 2:** *SETD2* mutated cases from our discovery and extension cohorts and published data

*Patient ID*	*SETD2 mutation cDNA change*	*SETD2 mutation protein change*	*Functional prediction (Polyphen2;SIFT)*	*Somatically acquired validated*	*dbSNP*	*MAF 1000 Genomes*	*Mutation Taster*	*Conserved*
*Discovery cohort*								
69	c.5863G>C	p.E1955Q		yes	rs761536283	—	P	*M. Musculus*
100	c.296A>G	p.D99G	-;D	yes	—	—	M	*M. Musculus*
255	c.4633C>A	p.Q1545K	P;T	ND	—	—	M	*D. Melano*
S21	c.3918G>A	p.W1306^a^	D;D	yes	—	—	M	*G. Gallus*

*Extension cohort (includes CLL8 cases)*								
149	c.2008G>A	p.E670K	P;D	yes	rs374976472	—	M	*G. Gallus*
88	c.5224A>C	p.M1742L	P;D	yes			M	*D. Rerio*
4273	c.148G>A	p.A50T	D;D	ND	rs191985301	0.020% (1/5008)	P	*M. Musculus*
4530	c.6885A>G	p.I2295M	B;D	ND	rs150476239	0.020% (1/5008)	M	*D. Melano*
4546	c.500C>T	p.P167L	B;-	ND	rs78682369	0.020% (1/5008)	P	Not conserved
4715	c.5666T>C	p.M1889T	P;D	ND	rs148097513	0.040% (2/5008)	M	*G. Gallus*
4172	c.5666T>C	p.M1889T	P;D	ND	rs148097513	0.040% (2/5008)	M	*G. Gallus*
4426	c.A1604G	p.N535S	B;T	ND	—	—	P	*M. Musculus*
4426	c.C265T	p.L89F	B,D	ND	—	—	P	*M. Musculus*
b266	c.5755-5781delGAAGAGGAAGAATTGCAGTCACAAC	p.EEEELQSQQ1919fs	-;-	yes	—	—	M	*M. Musculus*
b278	c.5411_5412delAC	p.L1804fs	-;-	yes	—	—	M	Partly conserved
b269	c.4727_4741delTCCTAGAATATTGTG	p.VLEYC1576del	-;-	yes	—	—	M	*M. Musculus*
b313	c.3568G>A	p.V1190M	B;T	yes	—	—	P	*M. Musculus*

*Landau et al.^b^and Puente et al.*								
b028	c.6433G>T	p.G2145^a^	-;D	yes	—	—	M	*M. Musculus*
b065	c.4973C>G	p.S1658^a^	-;T	yes	—	—	M	*M. Musculus*
a15	c.2225C>G	p.S742^a^	-;T	yes	—	—	M	Partly conserved
a^,b^141	c.6985C>T	p.Q2329^a^	-;T	yes	—	—	M	Not conserved
a,^b^141	c.7616A>T	p.K2539I	D;D	yes	—	—	M	*M. Musculus*
a177	c.3876-3877delGT	p.Y1293fs	-;-	yes	—	—	M	*M. Musculus*

Abbreviations: ND, not done, due to lack of germline material; PolyPhen2 prediction (B, benign; P, probably damaging; D, damaging); -, no prediction; SIFT prediction (D, damaging; T, tolerated; -, no prediction); MAF, minimal allele frequency in 1000 Genomes project; MutationTaster2 prediction (P, polymorphism; M, disease causing). Underlined text indicates an AID/APOBEC recognition motif. Mutation annotation was performed against COSMIC v 73 and no overlapping mutations were found.

a Cases included in Puente *et al.*

b Cases included in Landau *et al.*
