# Supplementary material for: Genomic disruption of the histone methyltransferase SETD2 in chronic lymphocytic leukaemia
Source: Leukemia. 2016 Jun 10;30(11):2179–86. doi: 10.1038/leu.2016.134 (PMC5023049; doi:10.1038/leu.2016.134)
Supplement: Supplementary Information [file leu2016134x1.docx]

# Genomic disruption of the histone methyltransferase SETD2 in chronic lymphocytic leukemia

Parker *et al*

## Supplementary Methods

### Patients

A discovery cohort comprised 261 patients and included 105 previously untreated patients randomized to the fludarabine and cyclophosphamide (FC) arm of the UK CLL4 trial and 146 untreated local patients with progressive disease. A second, extension cohort (previously untreated trial patients) included 201 tumor samples from two United Kingdom (UK) National Cancer Research Network (NCRN) clinical trials, ADMIRE [n= 93] and ARCTIC [n= 108], 156 tumor samples from patients randomized to the chlorambucil (CHL), fludarabine (F) and FC arms of the UK LRF CLL4 trial ^1^ , and 278 tumor and germline matched samples from the German CLL Study Group (GCSG), CLL8 Trial. ^2^ The third cohort comprised samples from 110 ultra-high risk patients randomized to the SCSG, CLL2O trial. All samples contained more than 80% tumor cells.

DNA was extracted from CLL B cells at trial randomization or the requirement for treatment (mean time from diagnosis to sampling 45.8 months, range 0 to 320 months for the non-trial patients). Matched germ-line DNA samples from *SETD2*-mutated cases was extracted from FACS-purified FITC-conjugated CD4+ T-cells or saliva extracted using the Oragene kit as per manufacturer’s instructions (DNA Genotek, Canada).

### Genome-wide microarray-based copy number analysis

DNA from 261 discovery and 110 ultra-high risk cases was amplified, labelled and hybridized to the Affymetrix SNP6.0 platform, aligned onto the human genome sequence (GRCh37) and analysed in Partek Genomics Suite (Partek Inc, Missouri, USA) as reported previously.^3-7^ DNA from 201 pre-treatment validation cases (ADMIRE and ARCTIC) was hybridized to the Illumina HumanOmni1-Quad and HumanOmniS-8 platforms according to manufacturer’s protocols found on registration at http://www.illumina.com/products, and data was processed using GenomeStudioV2009.2 (Illumina, San Diego, CA) ^8,9^. The total copy number data was analysed using Partek Genomics Suite (Partek Inc, Missouri, USA). We excluded from further analyses all losses and gains that had been noted previously in the Database of Genomic Variants (http://dgv.tcag.ca/dgv/app/home) or that were present in germ-line material from published CLL studies ^7,10^ and all copy neutral loss of heterozygosity (cnLOH) <2 Mb that did not extend to the telomere.

### Targeted re-sequencing and whole exome sequencing

For Haloplex analysis, subsequent to adapter trimming, the fastq sequence was aligned to the human reference genome sequence (hg19/GRCh37) using Novoalign (v2.08.02), followed by the removal of unmapped or poor-quality reads, and variant calling using samtools mpileup (v0.1.18), allowing a minimum read depth of 4 and a minimum phred scaled quality score of 20.

Initial alignment and variant calling analysis of the TruSeq data was performed with the BaseSpace online analysis tool (https://basespace.illumina.com). The sequence data were also analysed using the Stampy ^11^ and Platypus tools to screen for large insertions/deletions (http://www.well.ox.ac.uk/platypus). High confidence functional sequence variants were identified by removing any that failed the BaseSpace filter, had a quality score <60 and had no predicted functional consequence (intergenic, synonymous).

**Supplementary Methods Table 1. Genes screened for mutations in the discovery cohort**

|  | Gene Name | Chromosome | Start genomic position | End genomic position | Number of amplicons | Number regions targeted | Total coverage (%) |
| --- | --- | --- | --- | --- | --- | --- | --- |
| Methyltransferase, methyltransferase complex or SET domain genes | PRDM2 | 1 | 14057484 | 14149689 | 170 | 11 | 99.97 |
|  | SETDB1 | 1 | 150899243 | 150936850 | 197 | 22 | 100.00 |
|  | ASH1L | 1 | 155307440 | 155491320 | 313 | 29 | 99.99 |
|  | RBBP5 | 1 | 205057904 | 205091011 | 102 | 14 | 99.71 |
|  | SMYD2 | 1 | 214454587 | 214510137 | 97 | 12 | 99.99 |
|  | SMYD3 | 1 | 245912854 | 246670529 | 115 | 15 | 100.00 |
|  | SMYD1 | 2 | 88367373 | 88411648 | 119 | 11 | 99.96 |
|  | SETMAR | 3 | 4345044 | 4358940 | 49 | 4 | 92.37 |
|  | SETD5 | 3 | 9470612 | 9520306 | 251 | 26 | 99.59 |
|  | SETD2 | 3 | 47058572 | 47205424 | 310 | 27 | 99.46 |
|  | WDR82 | 3 | 52291495 | 52312387 | 71 | 9 | 100.00 |
|  | WHSC1 | 4 | 1902371 | 1980646 | 224 | 23 | 99.94 |
|  | SETD7 | 4 | 140417496 | 140477301 | 96 | 9 | 100.00 |
|  | PRDM9 | 5 | 23509132 | 23527892 | 78 | 10 | 77.52 |
|  | PRMT6 | 5 | 107599327 | 107600603 | 35 | 1 | 99.71 |
|  | PRDM6 | 5 | 122425699 | 122522905 | 67 | 7 | 96.63 |
|  | NSD1 | 5 | 176562094 | 176722470 | 285 | 26 | 99.54 |
|  | EHMT2 | 6 | 31847850 | 31865464 | 214 | 29 | 99.34 |
|  | PRDM1 | 6 | 106534418 | 106555371 | 123 | 9 | 100.00 |
|  | MLL5 | 7 | 104681389 | 104753827 | 211 | 27 | 98.65 |
|  | EZH2 | 7 | 148508706 | 148544400 | 146 | 21 | 93.32 |
|  | MLL3 | 7 | 151833906 | 152132881 | 598 | 62 | 95.12 |
|  | ASH2L | 8 | 37963058 | 37996599 | 133 | 17 | 99.98 |
|  | WHSC1L1 | 8 | 38133148 | 38205699 | 197 | 24 | 99.14 |
|  | SET | 9 | 131446164 | 131456953 | 62 | 11 | 98.86 |
|  | WDR5 | 9 | 137004989 | 137023125 | 119 | 13 | 100.00 |
|  | EHMT1 | 9 | 140513470 | 140741036 | 301 | 31 | 93.63 |
|  | SUV39H2 | 10 | 14920877 | 14944521 | 53 | 6 | 99.97 |
|  | MEN1 | 11 | 64571795 | 64577591 | 89 | 9 | 99.56 |
|  | SUV420H1 | 11 | 67925144 | 67957553 | 125 | 14 | 99.96 |
|  | EED | 11 | 85956261 | 85989577 | 83 | 13 | 100.00 |
|  | MLL | 11 | 118307217 | 118392897 | 477 | 39 | 98.07 |
|  | PRMT8 | 12 | 3490550 | 3702358 | 93 | 11 | 100.00 |
|  | MLL2 | 12 | 49415552 | 49449117 | 616 | 55 | 99.18 |
|  | SETD8 | 12 | 123868735 | 123892260 | 67 | 9 | 98.93 |
|  | SETDB2 | 13 | 50026019 | 50065995 | 135 | 14 | 99.67 |
|  | PRMT5 | 14 | 23389879 | 23398580 | 140 | 19 | 99.63 |
|  | SETD3 | 14 | 99866425 | 99932152 | 105 | 13 | 99.49 |
|  | SETD1A | 16 | 30970042 | 30995354 | 199 | 18 | 98.29 |
|  | SETD6 | 16 | 58549431 | 58552943 | 93 | 8 | 99.87 |
|  | PRMT7 | 16 | 68349872 | 68391137 | 145 | 17 | 99.99 |
|  | PRDM7 | 16 | 90124686 | 90142328 | 71 | 10 | 99.21 |
|  | SUZ12 | 17 | 30264255 | 30326032 | 84 | 16 | 84.02 |
|  | EZH1 | 17 | 40854539 | 40880978 | 136 | 19 | 95.00 |
|  | SETBP1 | 18 | 42281301 | 42643673 | 162 | 6 | 96.57 |
|  | CXXC1 | 18 | 47808952 | 47813969 | 109 | 15 | 99.98 |
|  | DOT1L | 19 | 2164173 | 2229801 | 298 | 31 | 97.50 |
|  | CARM1 | 19 | 10982368 | 11032443 | 123 | 16 | 97.93 |
|  | MLL4 | 19 | 36208910 | 36229468 | 336 | 37 | 98.15 |
|  | PRMT1 | 19 | 50183118 | 50191512 | 76 | 13 | 99.51 |
|  | SUV420H2 | 19 | 55853294 | 55858827 | 99 | 9 | 99.52 |
|  | SETD4 | 21 | 37408404 | 37431196 | 92 | 11 | 100.00 |
|  | PRMT2 | 21 | 48056853 | 48084296 | 138 | 12 | 99.81 |
|  | SUV39H1 | X | 48554200 | 48566280 | 73 | 9 | 97.43 |
| Genes recurrently mutated in CLL | NOTCH2 | 1 | 120457918 | 120612085 | 342 | 38 | 78.11 |
|  | SF3B1 | 2 | 198257016 | 198299733 | 217 | 27 | 99.61 |
|  | MYD88 | 3 | 38180142 | 38182787 | 51 | 5 | 99.79 |
|  | SI | 3 | 164697139 | 164793810 | 280 | 47 | 98.97 |
|  | KLHL6 | 3 | 183209704 | 183273451 | 93 | 7 | 99.99 |
|  | FAT4 | 4 | 126237556 | 126412933 | 503 | 18 | 99.86 |
|  | FBXW7 | 4 | 153244022 | 153332965 | 112 | 14 | 99.87 |
|  | DST | 6 | 56323792 | 56819395 | 1193 | 110 | 99.74 |
|  | KLHL7 | 7 | 23145635 | 23213927 | 104 | 17 | 99.10 |
|  | POT1 | 7 | 124462571 | 124540896 | 144 | 20 | 94.99 |
|  | BRAF1 | 7 | 140426283 | 140624513 | 141 | 21 | 99.41 |
|  | NOTCH1 | 9 | 139390512 | 139440248 | 376 | 34 | 97.89 |
|  | MUC2 | 11 | 1074891 | 1104273 | 500 | 50 | 92.13 |
|  | SF3B2 | 11 | 65819839 | 65836226 | 184 | 22 | 99.62 |
|  | BIRC3 | 11 | 102195230 | 102207843 | 77 | 9 | 98.29 |
|  | ATM | 11 | 108098341 | 108236245 | 562 | 62 | 96.30 |
|  | PMEL | 12 | 56347987 | 56364831 | 109 | 12 | 99.59 |
|  | CHD2 | 15 | 93444457 | 93567945 | 308 | 39 | 98.86 |
|  | HYDIN | 16 | 70841472 | 71264600 | 901 | 92 | 71.93 |
|  | TP53 | 17 | 7565246 | 7579922 | 118 | 14 | 95.54 |
|  | ZMYM3 | X | 70460755 | 70473115 | 210 | 24 | 99.46 |

Footnote: Haloplex designed using SureDesign (https://earray.chem.agilent.com/suredesign/)

### References

1. Catovsky D, Richards S, Matutes E, Oscier D, Dyer MJ, Bezares RF, et al. Assessment of fludarabine plus cyclophosphamide for patients with chronic lymphocytic leukaemia (the LRF CLL4 Trial): a randomised controlled trial. *Lancet* 2007; **370**:230-9.

2. Hallek M, Fischer K, Fingerle-Rowson G, Fink AM, Busch R, Mayer J, et al. Addition of rituximab to fludarabine and cyclophosphamide in patients with chronic lymphocytic leukaemia: a randomised, open-label, phase 3 trial. *Lancet* 2010; **376**:1164-74.

3. Parry M, Rose-Zerilli MJ, Gibson J, Ennis S, Walewska R, Forster J, et al. Whole exome sequencing identifies novel recurrently mutated genes in patients with splenic marginal zone lymphoma. *PLoS One* 2013; **8**:e83244.

4. Parker H, Rose-Zerilli M, Parker A, Chaplin T, Chen X, Wade R, et al. 13q deletion anatomy and disease progression in patients with chronic lymphocytic leukemia. *Leukemia* 2011; **25**:489-97.

5. Rose-Zerilli M, Forster J, Parker H, Parker A, Rodriguez A, Chaplin T, et al. ATM mutation rather than BIRC3 deletion and/or mutation predicts reduced survival in 11q-deleted chronic lymphocytic leukemia, data from the UK LRF CLL4 trial. *Haematologica* 2014; **99**:736-42.

6. Edelmann J, Saub J, Ibach S, Holzmann K, Tausch E, Bloehdorn J, et al. High Resolution Genomic Profiling of Primary “Ultra High Risk” and Refractory Chronic Lymphocytic Leukemia: Results from the CLL2O Trial *Blood (ASH Annual Meeting Abstracts)* 2014:3288.

7. Edelmann J, Holzmann K, Miller F, Winkler D, Bühler A, Zenz T, et al. High-resolution genomic profiling of chronic lymphocytic leukemia reveals new recurrent genomic alterations. *Blood* 2012; **120**:4783-94.

8. Knight SJ, Yau C, Clifford R, Timbs AT, Sadighi Akha E, Dréau HM, et al. Quantification of subclonal distributions of recurrent genomic aberrations in paired pre-treatment and relapse samples from patients with B-cell chronic lymphocytic leukemia. *Leukemia* 2012; **26**:1564-75.

9. Clifford R, Louis T, Robbe P, Ackroyd S, Burns A, Timbs AT, et al. SAMHD1 is mutated recurrently in chronic lymphocytic leukemia and is involved in response to DNA damage. *Blood* 2014; **123**:1021-31.

10. Ouillette P, Collins R, Shakhan S, Li J, Peres E, Kujawski L, et al. Acquired genomic copy number aberrations and survival in chronic lymphocytic leukemia. *Blood* 2011; **118**:3051-61.

11. Lunter G, Goodson M. Stampy: a statistical algorithm for sensitive and fast mapping of Illumina sequence reads. *Genome Res* 2011; **21**:936-9.
