# Supplementary material for: Genomic disruption of the histone methyltransferase SETD2 in chronic lymphocytic leukaemia
Source: Leukemia. 2016 Jun 10;30(11):2179–86. doi: 10.1038/leu.2016.134 (PMC5023049; doi:10.1038/leu.2016.134)
Supplement: Supplementary Figures [file leu2016134x2.docx]

**Supplementary Figure 1. Study cohort diagram.**


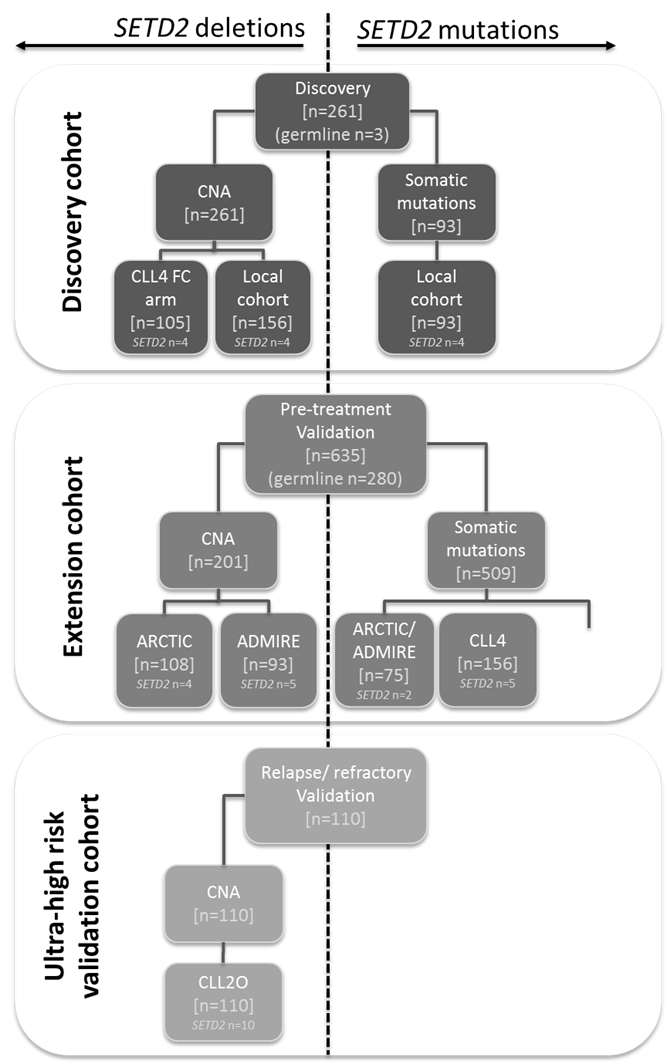


**Supplementary Figure 2**

**
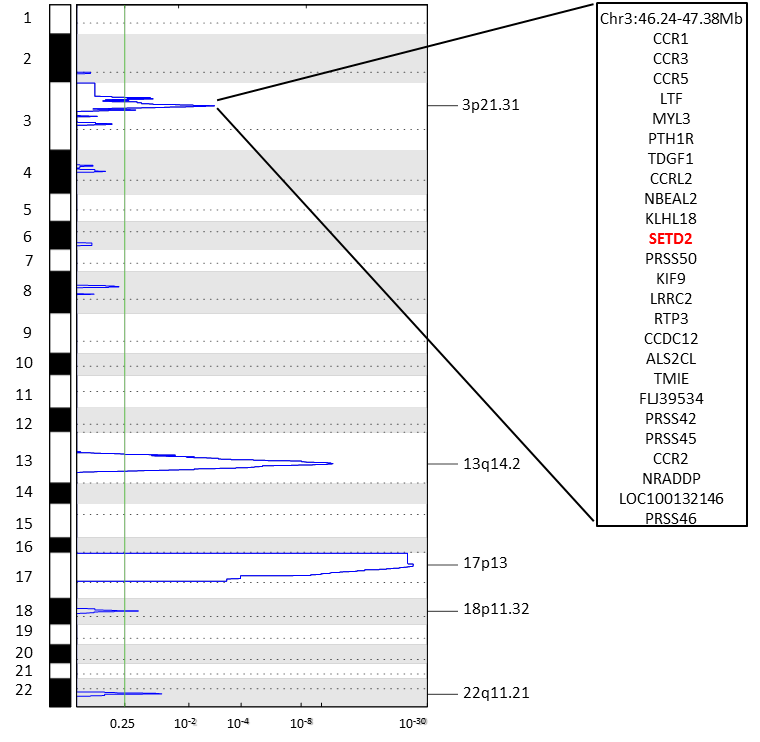
**

**Supplementary Figure 2. GISTIC analysis pf SNP 6.0 copy number data**

GISTIC 2.0 deletion plot from 39 del17p cases, including 15 cases with del3p (Mermel C, Schumacher S, et al. [Genome Biol.](http://www.ncbi.nlm.nih.gov/pubmed/21527027) 2011;12(4):R41)). The genome is oriented vertically from top to bottom, and GISTIC q-values at each locus are plotted from left to right on a log scale. The green line represents the significance threshold (q-value = 0.25). Wide peak boundaries are determined for each peak region (with greatest amplitude and frequency of alteration) to robustly identify the most likely gene targets in the region. A region on 3p21.31 (chr3:46245335-47382334) containing the *SETD2* gene and 24 others was identified in the wide peak boundary with a significant q-value (q=0.0014).

**Supplementary Figure3**


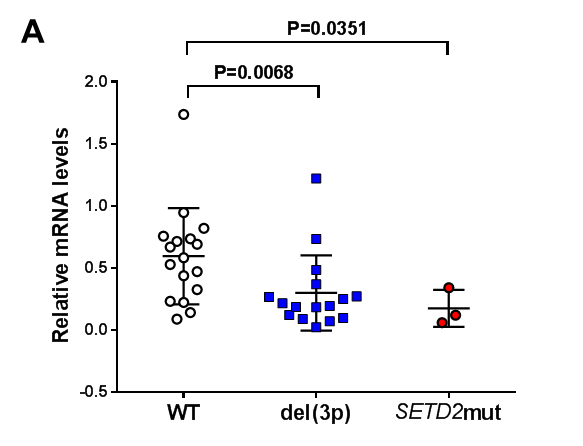


**Supplementary Figure 3. Analysis of SETD2 expression in wild-type and *SETD2* deleted and mutated CLL patients.** *SETD2* relative expression in wild-type (n=17), del(3p) cases (n=16) and *SETD2* mutated cases (n=3). P-values are indicated by bars above the data. 18S was used as housekeeping gene and levels of expression were normalized to normal B-cell mRNA.

**Supplementary Figure 4**

**Sub-clonal 3p deletions**

**(37%)**

**Clonal 3p deletions**

**(63%)**

**Any genome region**

**Genome region over SETD2 [del(3p)]**

**Genome region over**

**DLEU2 [del(13q)]**

**Genome region over**

**TP53 [del(17p)]**

**Normal copy number**

Genomic copy number

**Supplementary Figure 4.** Estimated 3p21 deletion clonality from SNP6.0 array derived segmentation mean values. The dot blot displays means for deleted regions only (≤1.7). *SETD2* [del(3p)], *DLEU2* [del(13q)] and *TP53* [del(17p)] are displayed as green, blue and red closed circles, respectively.

**Supplementary Figure 5**

**
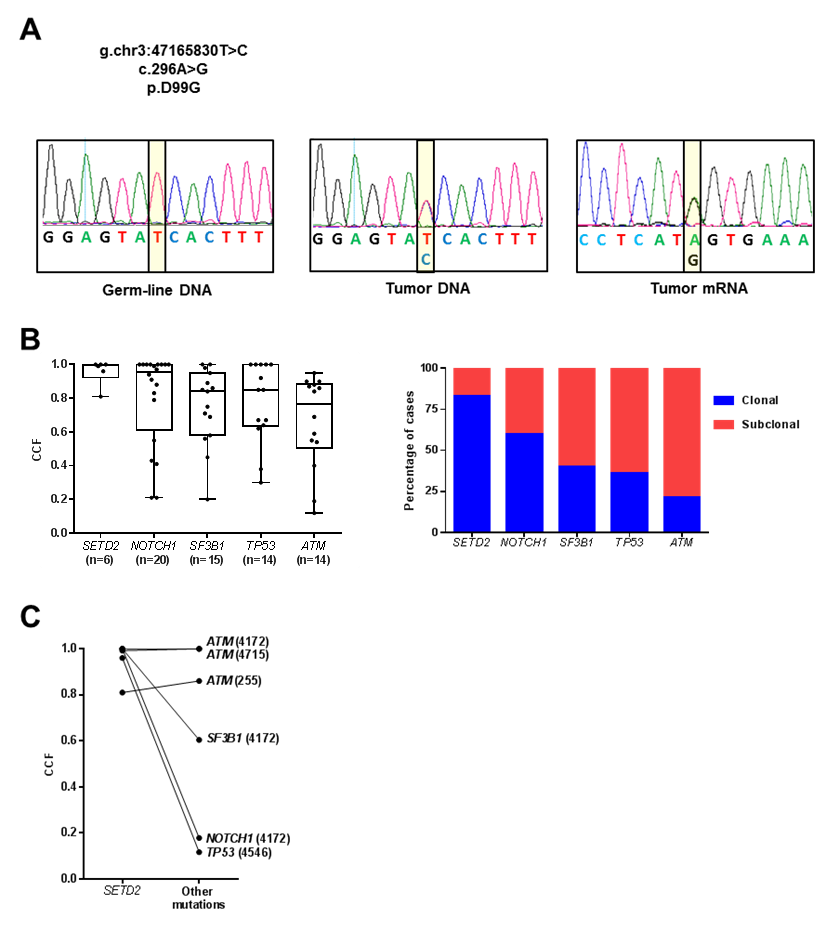
**

**Supplementary Figure 5. 5A. Example of the Sanger validation of one *SETD2* mutation. The variation is present on tumor DNA and mRNA but absent in germ-line DNA. 5B.** Analysis of the clonality for *SETD2* and other recurrently mutated genes on CLL. For each case the cancer cell fraction (CCF) is derived manually or with the ABSOLUTE algorithm. Somatically acquired non-validated mutations for *SETD2* are displayed. The number of mutations (n) for each gene in the analysis is shown (bottom). **5C.** Evolution of *SETD2*-mutant CLL. Estimated proportion of tumor cells harboring a *SETD2* mutation in comparison to other co-occurring mutations, displayed as an adjusted ratio of observed VAF divided by the 50% of the purity estimate derived from CD19+ FACS data. Sample IDs are in brackets.

**Supplementary Figure 5**

**
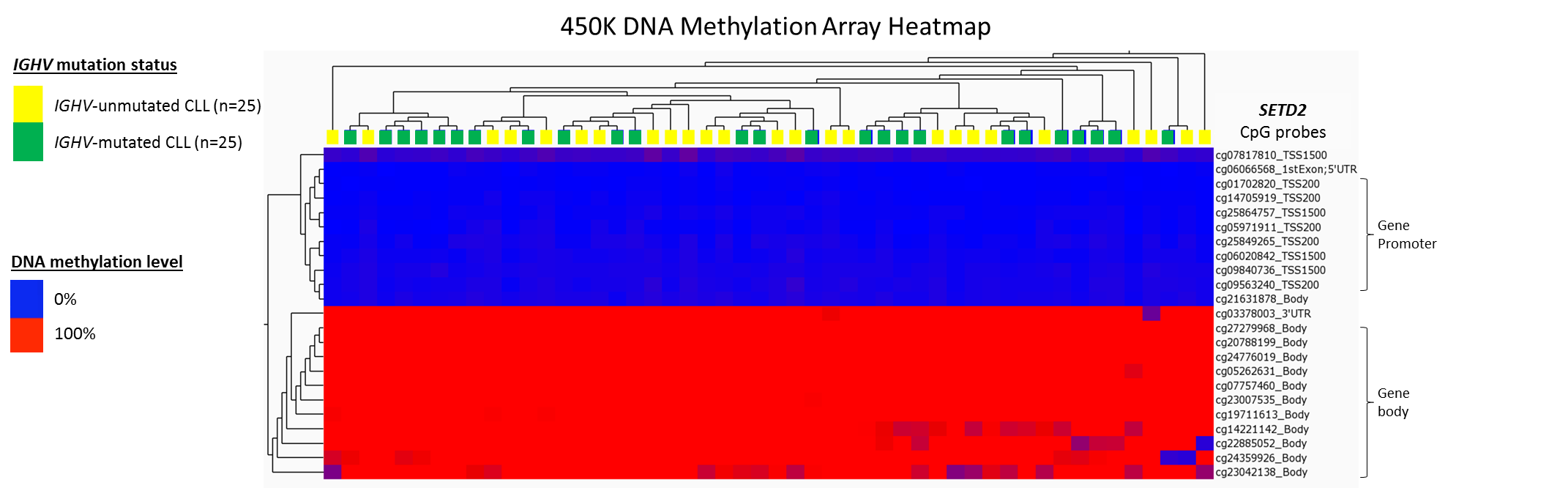
**

**Supplementary Figure 6: *SETD2* gene DNA methylation levels in *IGHV*-unmutated and –mutated CLL samples.** Heatmap (blue – low methylation, red-high methylation) showing methylation values (BMIQ-normalized, beta values) for prompter and gene-body CpG sites within *SETD2* gene loci (13 gene body and 10 promoter CpG sites). Unsupervised clustering analyses does not indicate a differential methylation signature for *IGHV* mutation status. We further compared the average methylation in promoter and gene-body (similarly to approach used in **[1]**) for patients with mutated and not mutated IGHV gene but did not observe statistical difference in methylation levels between the two groups (Two-sample Wilcoxon rank-sum (Mann-Whitney) test, promoter – P = 0.12, gene-body – P = 0.9). Hierarchical clustering also indicates characteristic for expressed genes methylation pattern with non-methylated promoter CpG sites and methylated gene body sites **[2]**.

**[1].** Assenov Y, Muller F, Lutsik P, Walter J, Lengauer T, Bock C: Comprehensive analysis of DNA methylation data with RnBeads. Nat Methods 2014, 11(11):1138-1140. [**2].** Lou S, Lee HM, Qin H, Li JW, Gao Z, Liu X, Chan LL, Kl Lam V, So WY, Wang Y et al: Whole-genome bisulfite sequencing of multiple individuals reveals complementary roles of promoter and gene body methylation in transcriptional regulation. Genome biology 2014, 15(7):408.

**Supplementary Figure 7**


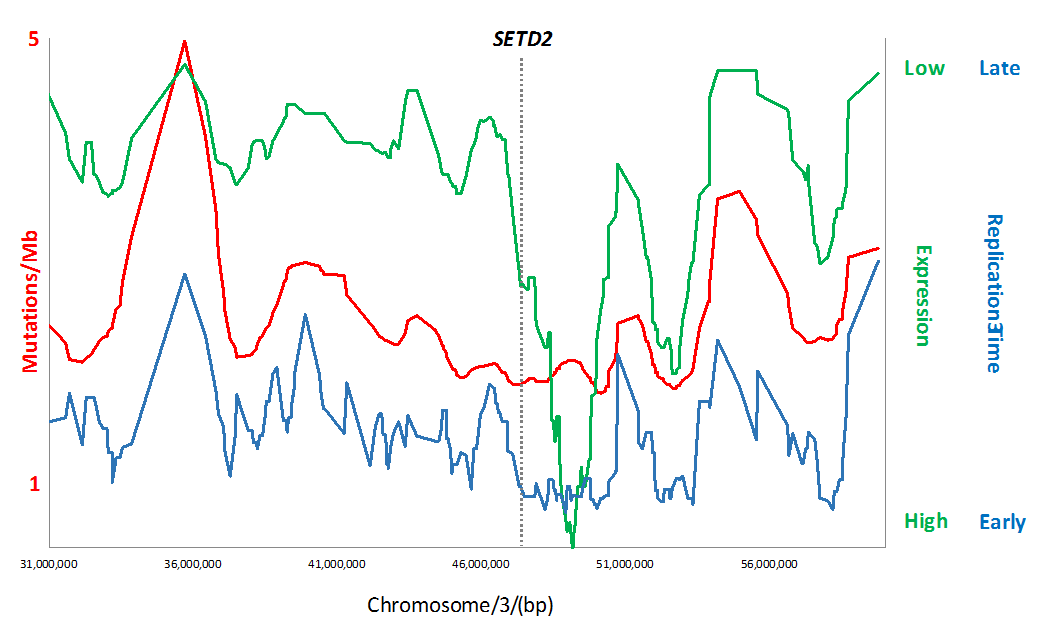


**Supplementary Figure 7: Chromosome 3 mutation rate, DNA replication time and expression level in the genomic location of the SETD2 locus.** Data from Lawrence et al. Mutational heterogeneity in cancer and the search for new cancer-associated genes. Nature 2013. Mutation rate, replication time and expression level plotted across a 30Mb region of chromosome 3 encompassing the *SETD2* gene. Red shows total non-coding mutation rate calculated from whole-genome sequences of 126 samples (excluding exons). Blue shows replication time. Green shows average expression level across 91 cell lines in the Cancer Cell Line Encyclopaedia determined by RNA sequencing. Note that low expression is at the top of the scale and high expression at the bottom, in order to emphasize the mutual correlations with the other variables.

Lawrence MS et al., Mutational heterogeneity in cancer and the search for new cancer-associated genes. Nature. 2013 Jul 11;499(7457):214-8

**Supplementary Table 1. Description of clinical trials used in the study.**

| **Clinical Trial** | **Phase** | | **Treatment naïve patients** | **Treatment arms** | **Total**  **N (%)** | **In this study**  **N (%)** |
| --- | --- | --- | --- | --- | --- | --- |
| **UK CLL4** | III | | Yes |  | 777 | 238 |
| **NCT00004218** |  | |  | Chlorambucil | 387 (50) | 81 (34) |
|  |  | |  | Fludarabine | 194 (25) | 27 (11) |
|  |  | |  | Fludarabine+Cyclophosphamide | 196 (25) | 130 (55) |
| **ARCTIC** | II | | Yes |  | 200 | 108 |
| **UKCRN ID 7136** |  | |  | Fludarabine+Cyclophosphamide+Rituximab | 100 (50) | 52 (48) |
|  |  | |  | Fludarabine+Cyclophospamide+Mitoxantrone +miniRituximab | 100 (50) | 56 (52) |
| **ADMIRE**  **UKCRN ID 6897** | II | | Yes |  | 215 | 93 |
|  |  | |  | Fludarabine+Cyclophospamide+Rituximab | * | 45 (48) |
|  |  | |  | Fludarabine+Cyclophospamide+Mitoxantrone +Rituximab | * | 48 (52) |
| **GCSG CLL8** | III | | Yes |  | 817 | 278 |
| **NCT00281918** |  | |  | Fludarabine+Cyclophosphamide | 409 (50) | 143 (51) |
|  |  | |  | Fludarabine+Cyclophosphamide+Rituximab | 408 (50) | 135 (49) |
| **SCSG CLL2O**  **NCT01392079** | II | Mixed | |  | 122 | 110 |
|  |  |  | | Alemtuzumab+dexamethasone induction followed by maintenance | 49 (37) | * |
|  |  |  | | Alemtuzumab+dexamethasone induction followed by allo-SCT | 33 (25) | * |

***No data available.**

**Supplementary Table 2. PCR primers for Sanger validation**

| Mutation | Exon | Material | Forward Primer Sequence | Reverse Primer Sequence |
| --- | --- | --- | --- | --- |
| p.A50T  p.D99G  p.P167L | 3 | DNA | ATTCTGCTATCACTGCTGGT | GACCAATGTTCAAAGGTGTT |
| p.E670K | 3 | DNA | TTTGCAGCAAGAAACCCTCG | GGGTCTCCAGCTCCATCAAA |
| p.W1306* | 3 | DNA | ACTTTCCAAAACAGGCCAGA | CGGACTGGTCTGAAAAATGG |
| p.Q1545K | 5 | DNA | CTAAGGAATCCCTTTGTGATGG | GTGAGCCAAGATCGTGCCAC |
| p.M1742L | 10 | DNA | AATCCCAGCTACTCAGGACG | GTGGTAGGTGGATGGAGAGC |
| p.M1889T  p.E1955Q | 12 | DNA | CAGATTATAAAGACTTTGGAACACTTG | CTCTTTGGGCTCTATTTCAGC |
| p.I2295M | 15 | DNA | ACAGTCTGTCAGTGTACAGCAGC | ATATTACCATGATGAAGGGTTCTCC |
| p.D99G | 3 | mRNA | CCAAAGGCACCAAAACAAAA | AGCAGTGGCCTGGATGTTAC |
| p.E670K | 3 | mRNA | AGAGAAAAGGCTGGGTCTCC | GGGGATAATTCCGATCCAGT |
| p.Q1545K | 5 | mRNA | AAGCGAATGCAGTGTGAGTG | CTAGGACAAAGGTGTTCGAAGG |
| p.E1955Q | 12 | mRNA | CTCTGATGCAACCAGTGAGC | GGCTCCTTTCACTCTCCACA |

**Supplementary Table 3. Quantitative Real-Time PCR primers**

| Gene | Forward Primer Sequence | Reverse Primer Sequence | UPL probe |
| --- | --- | --- | --- |
| 18S | GCAATTATTCCCCATGAACG | GGGACTTAATCAACGCAAGC | 48 |
| CCDC12 | GGAACTATGTCCCGGAGGAT | TCCTTCACCTTCTCCTCCAC | 35 |
| KIF9 | AGTTCCGGGTGGTTCTGAG | GGCTGCAAAGTCATTCCTGT | 4 |
| KLHL18 | GGGGAGCATGAATAGCAAGA | TAGCCCCCACAGACGTAGAT | 42 |
| NBEAL2 | TTGTGGCTGCTCTACTACGC | TGCTCTTCTTGAAGGCACCTA | 41 |
| SETD2 3' | GCCGCAGCAGTGACTACA | GCGGCAGATCCAAGAGATTA | 30 |
| SETD2 5' | AAAGAGCTCAAGGTGAAATAGCA | TTTGGACACCGAGAAGAACA | 60 |

## Supplementary Table 4. Description of minimally deleted or enhanced regions (MDRs/MERS) observed in at least 2% (n=6) of our discovery cohort, in order of prevalence.

| **Chromosome region** | **Frequency % (n=)** | **Start Genomic position (Mb)** | **End Genomic position (Mb)** | **Aberration Type** | **Size (Mb)** | **No. Genes** | **Gene content** |
| --- | --- | --- | --- | --- | --- | --- | --- |
| ^*^13q | 56 (146^(26)^) | 50.55 | 50.69 | Deletion | 0.14 | 4 | *DLEU1, TRIM13, KCNRG, DLEU2 (Mir16-1/Mir15A)* |
| ^*^11q | 26.4 (69) | 107.98 | 108.39 | Deletion | 0.41 | 6 | Includes *ATM* |
| ^*^17p MDR1 | 11 (29) | 0.18 | 7.88 | Deletion | 7.7 | 142 | Includes *TP53* |
| ^*^18p MDR1 | 6.1 (16) | 2.45 | 2.87 | Deletion | 0.42 | 2 | *NDC80, SMCHD1* |
| ^*^2p | 5.7 (15) | 58.38 | 61.92 | Gain | 3.54 | 8 | Includes *BCL11A, REL* |
| ^*^9q | 2.6 (7) | 70.96 | 79.11 | Deletion | 8.15 | 24 | many |
| ^*^20q | 2.6 (7^(1)^) | 3.84 | 12.70 | Deletion | 8.86 | 32 | many |
| ^*^14q | 2.3 (6) | 92.40 | 92.43 | Deletion | 0.03 | 1 | *FBLN5* |
| ^#^3p | 3 (8) | 46.96 | 47.39 | Deletion | 0.43 | 5 | *CCDC12, NBEAL2, SETD2, KIF9,KLHL18* |
| ^#^17p MDR2 | 11.5 (30) | 13.39 | 17.14 | Deletion | 3.74 | 23 | many |
| ^#^17p MDR3 | 9.2 (24) | 19.46 | 19.52 | Deletion | 0.06 | 1 | *SLC47A1* |
| ^#^8q MER2 | 5.3 (14) | 140.98 | 141.42 | Gain | 0.44 | 1 | *TRAPPC9* |
| ^#^8p | 4.6 (12) | 0.21 | 27.29 | Deletion | 27.08 | 113 | many |
| ^#^17p MDR4 | 3.8 (10) | 21.70 | 22.10 | Deletion | 0.38 | 1 | *FAM27L* |
| ^#^6q MDR3 | 3.4 (9) | 109.12 | 109.52 | Deletion | 0.4 | 3 | *ARMC2, SESN1* |
| ^#^4p | 3 (8^(1)^) | 11.14 | 11.63 | Deletion | 0.49 | 1 | *HS3ST1* |
| ^#^8q MDR | 2.3 (6) | 61.55 | 62.00 | Deletion | 0.45 | 1 | *CHD7* |

Footnote: (n) Number of cases with biallelic deletion of the MDR. ^*^Established/previously identified MDRs. ^#^Novel MDR/MERs. Trisomy 12 has been excluded in the table

**Supplementary Table 5. *SETD2* deleted cases from our discovery, extension and ultra-high risk cohorts**

|  | Patient ID | *SETD2* deletion Breakpoints ^1^ |
| --- | --- | --- |
| **Discovery cohort** | 258 | 46150516-50033424 |
|  | 197 | 46726524-50127996 |
|  | 103 | 46759996-49667692 |
|  | 193 | 45085712-53633824 |
|  | 286 | 60000-81006870 |
|  | 328 | 18336700-47399900 |
|  | 293 | 46700452-48712064 |
|  | 5 | 46962996-47416996 |
| **Extension cohorts** | 201 | 47061719-48517317 |
|  | 28 | 47124580-49955568 |
|  | 318 | 46422217-48228467 |
|  | 340 | 46702614-48727968 |
|  | 304 | 45638832-49111296 |
|  | 40 | 44451783-47362078 |
|  | 43 | 60000-83628960 |
|  | 268 | 60333-81373751 |
|  | 327 | 46700452-48712064 |
| **Ultra-high risk cohort** | 4 | 60000-80318681 |
|  | 005 | 45815388-47591674 |
|  | 33 | 46858822-50255974 |
|  | 39 | 18461075-52592025 |
|  | 41 | 39763121-52726591 |
|  | 55 | 33061850-49405560 |
|  | 68 | 60000-87218990 |
|  | 70 | 46266964-47405845 |
|  | 75 | 18076545-53731898 |
|  | 78 | 60000-112975611 |

Footnote. ^1^ Genome positions correspond to Hg19 version.

## Supplementary Table 6. Description of Minimally Deleted Regions (MDRs) observed in patients with chromosome 3 chromothyripsis (n=7).

| **Start Genomic position (Mb)** | **End Genomic position (Mb)** | **Frequency**  **(n=)** | **Size (Mb)** | **No. Genes** | **Gene content** |
| --- | --- | --- | --- | --- | --- |
| 4.77 | 9.32 | 4 | 4.5 | 11 | Includes *MIR4790, RAD18* |
| 21.40 | 21.87 | 6 | 0.47 | 1 | *ZNF385D* |
| 29.68 | 30.00 | 6 | 0.31 | 1 | *RBMS3* |
| 32.45 | 33.06 | 4 | 0.6 | 12 | Includes *IGBP1, CCR4* |
| 35.62 | 37.39 | 6 | 1.7 | 9 | Includes *MIR128-2, GOLG4* |
| 46.27 | 47.37 | 7 | 1.1 | 13 | ***SETD2*** |
| 47.80 | 49.11 | 5 | 1.3 | 53 | Many including *ATRIP* |
| 59.94 | 62.28 | 6 | 2.3 | 3 | *FHIT,MIR548BB,PTPRG* |
| 72.91 | 73.54 | 5 | 0.62 | 4 | *GXYLT2, PPP4R2* |
